# Complementary feeding and nutritional status of infants on cow’s milk proteins elimination diet

**DOI:** 10.1590/1984-0462/2022/40/2020429IN

**Published:** 2022-05-27

**Authors:** Érika Ozela Augusto, Vânia Guimarães Bonucci, Rafaela Valente Cardoso, Mauro Batista de Morais

**Affiliations:** a Universidade Federal de São Paulo, São Paulo, SP, Brasil.; b Unidade Básica de Saúde de Fátima, Belém, PA, Brasil.

**Keywords:** Milk hypersensitivity, Complementary feeding, Nutritional status, Infant, Milk substitutes, Hipersensibilidade a leite, Alimentação complementar, Estado nutricional, Lactentes, Substitutos do leite

## Abstract

**Objective:**

To evaluate the diet and nutritional status of infants on an elimination diet of cow’s milk proteins.

**Methods:**

Observational and cross-sectional study that compared: Infants on a cow’s milk protein elimination diet (n=60) assisted at a hypoallergenic formula distribution unit and a control group of same age and gender without dietary restrictions (n=60). Age ranged from 6 to 24 months. The diet was evaluated using the 24-hour food survey and weight and height were measured.

**Results:**

The macronutrient intake of both groups reached nutritional recommendations. The proportions of infants in the group of elimination of cow’s milk proteins with insufficient intake were lower, compared to controls, for iron (13.3 and 31.7%; p=0.029), zinc (5.0 and 18.3%; p=0.047), and vitamin D (25.0 and 71.7%; p<0.001). The hypoallergenic formula contributed to a greater supply of nutrients than dairy foods for the control group. Between 12 and 24 months, the number of infants on a restriction diet who never consumed meat, fish, cereals, and eggs was higher than in the control group (p<0.05). The length-age Z scores in infants on a cow’s milk protein elimination diet (-0.4±1.6) were lower (p=0.039) than in the control group (+0.2±1.3).

**Conclusions:**

The diet of infants with exclusion of cow’s milk protein was adequate despite the delay in the introduction of some complementary foods. Infants on an elimination cow’s milk protein diet showed lower linear growth without weight deficit.

## Resumo

**Objetivo:**

Avaliar a dieta e o estado nutricional de lactentes em dieta de exclusão das proteínas do leite de vaca.

**Métodos:**

Estudo observacional, transversal, que comparou: lactentes em dieta de exclusão das proteínas do leite de vaca (n=60) atendidos em unidade de dispensação de fórmulas hipoalergênicas e lactentes sem restrições alimentares (n=60), de mesma idade e sexo (grupo controle). A idade variou de seis a 24 meses. A dieta foi avaliada com o emprego do inquérito alimentar e foram mensurados o peso e a estatura.

**Resultados:**

A ingestão de macronutrientes foi adequada em ambos os grupos. No grupo em dieta de exclusão das proteínas do leite de vaca, as proporções de lactentes com ingestão insuficiente foram menores em relação aos controles, para ferro (13,3 e 31,7%; p=0,029), zinco (5,0 e 18,3%; p=0,047) e vitamina D (25,0 e 71,7%; p<0,001). A fórmula hipoalergênica contribuiu com maior oferta de nutrientes do que os alimentos lácteos para o grupo controle. Entre 12 e 24 meses, o número de lactentes em dieta de exclusão que nunca consumiram carne bovina, peixe, cereais e ovo foi maior do que no grupo controle (p<0,05). Os escores Z de comprimento-idade nos lactentes em dieta de exclusão das proteínas do leite de vaca (-0,4±1,6) foram menores (p=0,039) do que no grupo controle (+0,2±1,3).

**Conclusões:**

A dieta de lactentes em exclusão do leite de vaca foi adequada apesar do atraso na introdução de alguns alimentos. Lactentes em dieta de exclusão apresentaram menor crescimento linear não acompanhado de déficit ponderal.

## INTRODUCTION

Food allergy is an adverse reaction resulting from an abnormal and reproducible immune response, triggered by the ingestion of one or more foods. Cow’s milk protein allergy (CMPA) is the most frequent type of food allergy in the first year of life, with an estimated prevalence between 2 and 3%.^1,2,3,4^

The treatment of CMPA is based on the exclusion of the allergenic protein and on the prescription of a substitute diet that fully meets the patient’s nutritional needs. The restriction should be continued until the patient develops oral tolerance, usually after the first or second year of life.^1,2^

As with healthy infants, patients with CMPA should start complementary feeding in the sixth month of life. No restrictions should be made, except for foods containing cow’s milk proteins.^1,2,5,6^

It is well established that the delay in the introduction of potentially allergenic foods does not provide a beneficial effect for the infant undergoing CMPA treatment. Therefore, the introduction of complementary foods must follow the same recommendations as those for healthy children,^1,2,5,6^ provided that this process must be carried out more carefully, observing any possible reactions for each new food added to the diet.^1,2^

It is also essential that the restriction meets all nutritional needs, not only due to the high growth rate observed in this age group, but also due to the evidence that indicates nutritional impairment in patients with CMPA.^7–11^

Although scarce, studies carried out in Brazil^9,10^ and in other countries^7,8,11^ have shown that anthropometric deficits are frequently observed in infants with CMPA. However, the determining factors of nutritional deficit are not well defined. Theoretically, it can be established before the beginning of the restriction diet and be due to the cow’s milk protein itself or the persistence of the inflammatory state resulting from accidental or voluntary ingestion of the allergenic protein, or even from qualitative and/or quantitative inadequacies of the exclusion diet. Of course, these mechanisms can act alone or accompanied.^7,9,11,12^

Considering that there is little information about the restriction diet for the treatment of CMPA, especially regarding the characteristics of complementary feeding, this study was carried out with the aim of evaluating the diet and nutritional status of infants on a cow’s milk protein elimination diet compared to infants with no dietary restrictions.

## METHOD

In this cross-sectional study, two groups of infants matched by gender, age group, and economic classification were compared:

1. study group on cow’s milk protein elimination diet;
2. a control group consisting of infants with no dietary restrictions.

The project was approved by the Research Ethics Committee of *Universidade Federal de São Paulo*, under number 081783/2015. Informed consent was obtained, in writing, from those responsible for the infants included in the study. Data collection was carried out between February 2016 and August 2018.

Sixty children, aged between six and 24 months, suspected or diagnosed with CMPA and who were on an elimination diet of cow’s milk and dairy products, were included. Infants were consecutively included in the Municipal Health Unit of Fátima (Belém, Pará). This public care unit is the only one in the city where hypoallergenic formulas for infants with CMPA are dispensed. Inclusion criteria were age between six and 24 months and being in an elimination diet by CMPA.

Exclusion criterion was the presence of other diseases in which special diets are used (Crohn disease, celiac disease, short bowel syndrome) and/or diseases that prevent the administration of food exclusively by mouth, such as those with neurological impairment.

For the control group, the same inclusion and exclusion criteria were considered, except for the type of diet, that is, no food restrictions. For pairing, the same gender, economic status, and age range (same quarter of life) were respected in relation to infants admitted to the group on cow’s milk and dairy products restriction diet. Thus, the control group consisted of 60 infants who received a diet without food restriction, paired with the group on a restriction diet. The infants who constituted the control group were recruited from the well baby clinic of *Maternidade Saúde da Criança*, in Belém, Pará.

In estimating the sample size, a power of 0.8 and an α error of 5% were used. The two groups were considered to have a minimum difference between the mean weight-age and length-age Z scores of 0.6, with a standard deviation of 1.0, as observed in previous research.^9,10^ The minimum number of 45 infants for each group was calculated with the use of The SigmaPlot 11.2 software (San Jose, CA, USA).

A standardized form developed by the researchers was used to obtain clinical, demographic, and nutritional information. For the economic evaluation, the criterion proposed by the Brazilian Association of Research Companies (*Associação Brasileira de Empresas de Pesquisas* – ABEP) was adopted.^13^ The information was obtained from the parents during an interview with one of the researchers.

For the assessment of food consumption, the 24-hour recall survey was used.^14^ To standardize the size of the portions, photos of the food portions were used.^15^ During the interview, models of utensils (cups, cutlery, plates, baby bottles) were used to set the amount of food. The age at which food was introduced and the current consumption of certain items were also questioned, regardless of whether or not they were mentioned in the 24-hour recall survey. To estimate breast milk intake, the multiple linear regression model proposed by Drewett et al. for supplemented breastfeeding was used: Y=755.0–0.48X’–0.59X” , where Y is the estimate of breast milk intake, X’ is the age in days and X” is the consumption of complementary foods in kilocalories.^16^ The calculations of nutrients in the diet were performed using the Avanutri online software (AVANUTRI, Três Rios, RJ, Brazil). For the analysis of nutritional adequacy, the recommended daily intake (dietary reference intakes – DRI) was adopted.^17,18,19,20^

Weight and length were measured according to the recommendations of the Food and Nutritional Surveillance System of the Ministry of Health.^21^ To measure weight, an electronic pediatric scale, model 109-E, by Welmy (Santa Bárbara d’Oeste, Brazil), with a capacity of 15kg and an accuracy of 5g. To measure the length, a portable horizontal infantometer, model Inf-100, by Balmak (Santa Bárbara d’Oeste, Brazil), with 1mm precision was used.

The Z scores for weight/age, height/age, and weight/height were calculated using the WHO Anthro software (version 3.2.2, 2011), which uses the reference values proposed by the World Health Organization.^22^

Data were tabulated in a Microsoft Excel 2011 spreadsheet, version 14.7.3 (for Mac). Statistical analysis was performed using the SigmaPlot software, version 11 (Systat Software, San Jose, CA, USA). A significance level of 5% was considered in all analyses.

## RESULTS

The results in Table 1 show that the procedure for matching the groups according to age, gender, and economic classification was adequate. Infants on an elimination diet of cow’s milk and dairy products had a shorter duration of exclusive breastfeeding and a higher frequency of antibiotic use in the first semester of life. In both groups there was a high frequency of cesarean sections and a family history of allergy. In the majority (71.7%; 43/60) of infants, clinical manifestations began in the first trimester of life. The median (25^th^ and 75^th^ percentiles) of the number of clinical manifestations motivating the elimination diet indication was 7.0 (5.0 and 9.5). The most common were gastrointestinal manifestations, which were present in the following percentages of the 60 infants: abdominal pain/colic (66.7%), diarrhea (61.7%), vomiting or regurgitation (60%), presence of blood in the feces (58.3%), and abdominal distension (58.3%). Next, cutaneous manifestations occurred, with a predominance of atopic dermatitis in 61.7% of infants. Irritability, refusal to eat and difficulty in gaining weight/height occurred, respectively, in 48.3, 41.7, and 38.3% of the infants. One of them had a history of anaphylaxis. The median age at start of the elimination diet was 3.5 months (25^th^ and 75^th^ percentiles: 1.5 and 6.0 months).

**Table 1.** Demographic data, history and anthropometric indicators (Z score) of the group on cow’s milk protein exclusion diet and the control group

	Group on cow’s milk restriction diet (n=60)	Control group (n=60)	p-value
Gender			
Male^a^	27 (45.0%)	28 (46.7%)	1.000
Female^a^	33 (55.0%)	32 (53.3%)	
Age (months)^b^	12.0 (10.0–15.0)	12.0 (10.0–15.0)	0.971
Economic status (ABEP)			
A and B^a^	42 (70.0%)	42 (70.0%)	1.000
C, D, and E^a^	18(30.0%)	18(30.0%)	
Family history of allergy^a^	40 (66.7%)	52 (86.7%)	0.495
Cesarean delivery^a^	54 (90.0%)	52 (86.0%)	0.776
Premature birth^a^	11 (18.3%)	5 (8.3%)	0.179
Use of antibiotics in the first semester of life^a^	30 (50.0%)	9 (15%)	<0.001
Duration of exclusive natural breastfeeding (months)^b^	1.0 (0.0–4.0)	6.0 (5.0–6.0)	<0.001
Anthropometric indicators (Z score)			
Weight/Age^c^	+0.2±1.1	+0.2±1.0	0.986
Length/Age^c^	-0.4±1.6	+0.2±1.3	0.039
Weight/Length^c^	+0.5±1.3	+0.2±1.1	0.100

^a^Number, percentage, chi-square test; ^b^Median, 25^th^ and 75^th^ percentiles, Mann-Whitney test; ^c^Mean, standard deviation, Student’s *t*-test; ABEP: Brazilian Association of Research Companies (Associação Brasileira de Empresas de Pesquisas).

With regard to anthropometric indicators (Table 1), it was observed that the Z scores for weight/age and weight/length, expressed as continuous variables, were similar in both groups. In turn, the length/age Z scores were lower in the exclusion diet group. On the other hand, the proportions of infants with Z scores below the limit of -2.0 standard deviations of weight/age, length/age, and weight/length were, respectively: 0.0, 16.7, and 1.7% in the exclusion diet group and 3.3, 6.7, and 5.0% in the control group. The statistical study showed no difference between groups.

At the time of the study, in the elimination diet group, 41 (68.3%) infants were receiving extensively hydrolyzed protein formula, 16 (26.7%) were receiving free amino acid formula, and three (5.0%) were receiving formula based on isolated soy protein. Of the 60 infants, 25 (41.7%) excluded, in addition to milk, one or more foods from the diet, mainly beef (15.0%, 9/60), eggs (13.3%, 8/60), and soybeans (11.7%, 7/60).

Estimates of daily intake of energy and lipids were lower in the group on a cow’s milk protein elimination diet. On the other hand, iron, zinc, and vitamin D intakes were higher. There was no statistically significant difference in the intake of proteins, carbohydrates, calcium, vitamin A, and vitamin C (median values, dispersion and p values available from the corresponding author). Although statistically significant differences were found in the estimation of energy and lipids in relation to what was recommended by the DRI, all infants in both groups reached their individual daily requirements of energy, carbohydrates, proteins, and lipids, considering age and gender.

Table 2 shows the number and percentage of infants who had lower food intakes than that recommended by the DRI for calcium, phosphorus, iron, sodium, potassium, zinc, vitamins A, C, and D. The group on cow’s milk exclusion diet presented, compared to the control group, a lower percentage of infants with inadequate dietary intake of iron, zinc, and vitamin D in relation to DRI.

**Table 2.** Intake lower than the recommended daily intake of calcium, phosphorus, iron, sodium, potassium, zinc, and vitamins A, C, and D in the group on cow’s milk protein restriction diet and in the control group

	Group on cow’s milk restriction diet (n=60)	Control group (n=60)	p-value^a^
Calcium			
Lower than the DRI (270^b^/500^b^mg/day)	4 (6.7%)	5 (8.3%)	1.000
Phosphor			
Lower than the DRI (275^b^/460^c^mg/ day)	8 (13.3%)	9 (15.0%)	1.000
Iron			
Lower than the DRI (7^c^/11^c^mg/day)	8 (13.3%)	19 (31.7%)	0.029
Zinc			
Lower than the DRI (3^b,c^mg/day)	3 (5.0%)	11 (18.3%)	0.047
Vitamin A			
Lower than the DRI (500^b^/300^c^mg/day)	2 (3.3%)	3 (5.0%)	1.000
Vitamin C			
Lower than the DRI (50^b^/15^c^mg/day)	0 (0.0%)	2 (3.3%)	0.476
Vitamin D			
Lower than the DRI (5^b^mg/day)	15 (25.0%)	43 (71.7%)	<0.001

100% of infants in both groups met the estimated energy, protein, lipid, and energy requirements. ^a^Number, percentage, chi-square test; ^b^AI, adequate intake; ^c^RDA, recommended dietary allowances; DRI: dietary reference intakes.

Graphic 1 shows the percentage, in relation to the total daily intake, of energy and nutrients from hypoallergenic formulas in the group treated with CMPA or from breast milk and bottles with CMP in the control group. In both groups, hypoallergenic formulas or breast milk and bottles represent an important source of energy and nutrients. The percentages of lipids, iron, phosphorus, sodium, zinc, vitamins A, C, and D provided by hypoallergenic formulas were higher (p<0.05) in the group on CMP restriction diet than those offered by milk breast feeding and bottles in the control group.

**Graphic 1.**
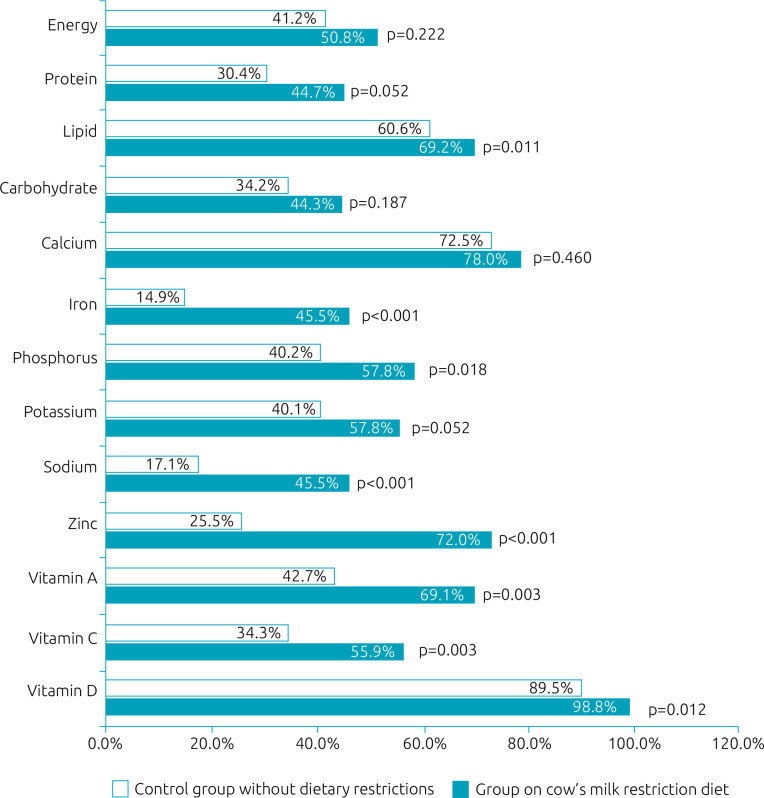
Percentage contribution of dairy sources (hypoallergenic formula or breast milk, infant formula, whole milk) in relation to total energy and nutrient intake in the two groups of infants studied

Table 3 shows the age of introduction and the foods that have not yet been introduced into the infants’ diet. For this analysis, only infants aged between 12 and 24 months were considered. The introduction of water or tea occurred at a younger age in the group on cow’s milk restriction diet, but the introduction of egg occurred later in this group when compared to the control group. It is also observed that, in the second year of life, the proportion of children who were not offered meat, fish, cereals, oilseeds, and eggs was higher in the group on the restriction diet. Regarding the consumption of foods not recommended for infants, it was found that in both groups there was consumption by infants of sausages, sweets, soft drinks, artificial fruit juice, and coffee. Coffee consumption was mentioned more frequently (p<0.05) in infants in the control group.

**Table 3.** Age of introduction, foods that have not yet been introduced in the diet, and foods that are unsuitable for feeding infants aged between 12 and 24 months in the group on cow’s milk protein restriction diet and in the control group

	Group on cow’s milk restriction diet (n=34)	Control group (n=33)	p-value
Age of food introduction (months)			
Water or tea^a^	3.5 (1.0–6.8)	5.0 (4.0–6.0)	0.016
Natural fruits^a^	6.0 (5.0–6.0)	6.0 (5.0–6.0)	0.638
Vegetables and tubers^a^	6.0 (5.8–6.3)	6.6 (6.0–6.0)	0.263
Egg^a^	9.0 (7.0–10.5)	7.0 (6.0–8.0)	0.005
Beef meat^a^	6.0 (6.0–9.0)	6.0 (6.0–7.0)	0.356
Chicken meat^a^	6.0 (6.0–7.0)	6.0 (6.0–7.0)	0.383
Fish meat^a^	6.0 (6.0–10.0)	7.0 (6.0–8.0)	0.993
Number (%) of infants who have never consumed the following foods:			
Beef meat^b^	5 (14.7%)	0 (0.0%)	0.005
Chicken meat^b^	0 (0.0%)	0 (0.0%)	1.000
Fish meat^b^	8 (23.5%)	1 (3.0%)	0.027
Cereals (wheat)^c^	14 (41.2%)	2 (6.1%)	0.002
Oilseeds^c^	29.0 (85.3%)	16 (48.5%)	0.003
Egg^b^	5 (14.7%)	0 (0.0%)	0.005
Number (%) of infants who consumed the following inappropriate foods for their age group			
Embedded foods^b^	6 (17.6%)	1 (3.4%)	0.106
Coffee^c^	10 (29.4%)	27 (81.2%)	<0.001
Sweets^c^	24 (29.4%)	11 (33.3%)	0.934
Soft drinks^b^	10 (20.6%)	2 (6.1%)	0.150
Artificial juice^c^	6 (17.6%)	5 (15.1%)	0.957

^a^Median, 25^th^ and 75^th^ percentiles, Mann-Whitney test; ^b^Number, percentage, Fisher’s exact test; ^c^Number, percentage, chi-square test;^4^Only children over one year of age were included;^5^To calculate the age at which foods were introduced, only infants who had consumed them were counted.

## DISCUSSION

In the present study, carried out in Belém, Pará, Northern Brazil, it was observed that infants undergoing treatment for CMPA reached the DRI not only for energy but also for proteins, lipids, and carbohydrates. On the other hand, the proportion of infants being treated for CMPA with adequate intake, according to the DRI, of iron, zinc, and vitamin D was higher than in the control group. There was a delay in the introduction of meat, fish, wheat, oilseeds, and eggs to infants undergoing treatment with CMPA. Hypoallergenic formulas in the exclusion diet group represented more important sources of nutrients in relation to total intake than breast milk and bottles for the control group. Finally, infants with CMPA showed lower values of height-for-age z scores. This set of objective information is a topic that has been little explored in the literature and has great practical relevance in nutritional assistance to patients undergoing treatment for CMPA.

In the group of infants being treated for CMPA, there was a linear growth deficit, characterized by lower values of the length-age Z scores. This result is in agreement with studies carried out previously in Brazil^9,10^ and in other countries.^7,8,11^ Lower height-age values may represent a sequelae of the CMPA active phase before the start of the allergenic protein restriction diet, that is, consequence of previous nutritional deficiency. However, to date, the mechanism of growth restriction in CMPA is still not completely understood. Evidence suggests the interaction of several factors, including deficiency in nutrient intake, before and after initiation of the elimination diet; inflammatory process characteristic of the allergic process; lower bioavailability of nutrients; and greater nutritional requirements. A recent review article critically analyzed the existing information on the relationship between food allergy, elimination diet, and growth deficit.^12^ It is highlighted that, in food allergy, there may be persistence of a subclinical inflammatory state associated with abnormalities in intestinal permeability, similar to what was observed in atopic individuals.^12^ This abnormality was described more than 20 years ago in a study^7^ carried out in Finland, in which infants, even on an appropriate restriction diet, presented lower linear growth compared to the control group. These intestinal abnormalities are essentially similar to those observed in environmental enteric dysfunction that affects children living in poverty and who predominantly present a deficit in linear growth even without expressing clinical gastrointestinal manifestations.^23^ However, environmental enteric dysfunction occurs in compatible socioeconomic strata with poverty and, therefore, it would not explain the findings in Finnish infants or in the infants included in the present study, who belonged, for the most part, to the highest socioeconomic classes in the country.

Regarding diet, the present study showed that both infants undergoing treatment for CMPA and the control group had an adequate intake of macronutrients in relation to DRI. In turn, the proportion of infants with consumption below the DRI for iron, zinc, and vitamin D was lower in the group of infants undergoing treatment for CMPA. It should be noted that patients undergoing treatment for CMPA were recruited from a public health unit that provides nutritional assistance and free dispensing of hypoallergenic formulas. It was also observed that the contribution of hypoallergenic formulas for infants on a restriction diet was important to provide a nutritionally adequate diet. Regarding vitamin D, a recent study carried out in Recife, Northeast Brazil, showed that the occurrence of low blood levels of vitamin D is lower in infants with CMPA who receive hypoallergenic formula.^24^ These data reinforce the importance of using hypoallergenic formulas even after starting complementary feeding and throughout the second year of life.

Infants undergoing treatment for CMPA should start complementary feeding in the sixth month of life, similarly to infants without dietary restrictions. The only restriction are foods with CMP.^1,2^ However, the present study showed that a portion of infants on a cow’s milk protein elimination diet did not yet consume several foods in the second year of life, such as meat, fish, wheat, egg, and oilseeds, in disagreement with complementary feeding guidelines. In future projects, it will be important to assess the reasons why these foods were not included in the diet, that is, whether it was carried out due to recommendation of the health team or on the initiative of the family. It should be mentioned that, in a study carried out in Brazil, it was observed that a significant portion of physicians and nutritionists still believed that delaying the introduction of food could prevent the occurrence of allergic diseases,^25^ in disagreement with the guidelines and evidence on the subject.^1,2,26^ It should be noted that delayed food introduction may be associated with a higher risk of food allergy, such as to eggs, as recently observed.^26^ Another interesting aspect is that, except for coffee, the proportion of children who had ever consumed foods that were inappropriate for their age, such as sweets and soft drinks, were similar in infants undergoing CMPA treatment and those without dietary restrictions, contrary to the expectation that families of infants undergoing CMPA treatment were more careful about their diet. The supply of inappropriate foods for infants had already been registered in Belém do Pará^27^ and in other regions of Brazil.^28^

Among the limitations of the study, it should be taken into account that a convenience sample of a single city was studied, which makes it impossible to generalize the data to the universe of infants on a restriction diet. However, it is likely that the same dietary practices occur in other regions of Brazil, as well as in other countries. Another limitation stems from the impossibility of establishing a causal relationship between the cow’s milk elimination diet and CMPA, since not all infants in the restriction diet group had a confirmed diagnosis of food allergy. Therefore, the results of the present study support further research on this topic, using more comprehensive designs. In turn, the sample size was estimated based on the expectation of differences in anthropometric parameters, however, it allowed, with regard to dietary information, to find several statistically and nutritionally significant differences between the two groups studied.

Another aspect was the inclusion of a greater number of infants from families of social levels A and B, which is not in accordance with the socioeconomic distribution of the Brazilian population. This point is mitigated by the fact that allergic diseases are more common in higher socioeconomic levels. It is also possible that in the more privileged classes there is greater access to information related to health and attention to the occurrence of clinical manifestations of food allergy.

However, care must be taken to ensure that, in view of the principle of universality of the Unified Health System in Brazil, access is provided to all infants with CMPA to special formulas dispensing services. A strong point of the present study is the fact that it is one of the first, as far as we know, to evaluate the complementary feeding of infants with CMPA, providing support for the development of strategies to improve nutritional assistance for infants on a restriction diet for cow’s milk and dairy products.

In conclusion, infants undergoing treatment for CMPA show less linear growth even with an adequate restriction diet from a qualitative and quantitative point of view. Hypoallergenic formulas are more important sources of nutrients for infants undergoing CMPA treatment than for infants without dietary restrictions, and the proportion of infants with insufficient intake of iron, zinc, and vitamin D is smaller. In a proportion of infants undergoing treatment for CMPA, in relation to controls, there was a delay in the introduction of foods to which a greater potential for causing food allergy is attributed.
